# Drought as an emergent driver of ecological transformation in the twenty-first century

**DOI:** 10.1093/biosci/biae050

**Published:** 2024-07-10

**Authors:** Wynne E Moss, Shelley D Crausbay, Imtiaz Rangwala, Jay W Wason, Clay Trauernicht, Camille S Stevens-Rumann, Anna Sala, Caitlin M Rottler, Gregory T Pederson, Brian W Miller, Dawn R Magness, Jeremy S Littell, Lee E Frelich, Abby G Frazier, Kimberley T Davis, Jonathan D Coop, Jennifer M Cartwright, Robert K Booth

**Affiliations:** Conservation Science Partners, Truckee, California, United States; U.S. Geological Survey, Northern Rocky Mountain Science Center, Bozeman, Montana, United States; Conservation Science Partners, Truckee, California, United States; USDA Forest Service, Fort Collins, Colorado, United States; North Central Climate Adaptation Science Center and with the Cooperative Institute for Research in Environmental Sciences, University of Colorado, Boulder, Colorado, United States; School of Forest Resources at the University of Maine, Orono, Maine, United States; Department of Natural Resources and Environmental Management at the University of Hawai'i at Mānoa, Honolulu, Hawai'i, United States; Colorado Forest Restoration Institute in the Forest and Rangeland Stewardship Department at Colorado State University in Fort Collins, Colorado, United States; Division of Biological Sciences at the University of Montana, Missoula, Montana, United States; South Central Climate Adaptation Science Center, University of Oklahoma, Norman, Oklahoma, United States; U.S. Geological Survey, Northern Rocky Mountain Science Center, Bozeman, Montana, United States; U.S. Geological Survey, North Central Climate Adaptation Science Center, Boulder, Colorado, United States; U.S. Fish and Wildlife Service, Kenai National Wildlife Refuge, Soldotna, Alaska, United States; U.S. Geological Survey, Alaska Climate Adaptation Science Center, Anchorage, Alaska, United States; Department of Forest Resources at the University of Minnesota, Saint Paul, Minnesota, United States; Graduate School of Geography at Clark University, Worcester, Massachusetts, United States; Department of Ecosystem and Conservation Sciences at the University of Montana, Missoula, Montana, United States; Missoula Fire Sciences Laboratory, Rocky Mountain Research Station of the USDA Forest Service, Missoula, Montana, United States; Clark School of Environment and Sustainability, Western Colorado University, Gunnison, Colorado, United States; U.S. Geological Survey, Southeast Climate Adaptation Science Center, Raleigh, North Carolina, United States; Earth and Environmental Science Department at Lehigh University, Bethlehem, Pennsylvania, United States

**Keywords:** climate change, disturbance, drought, ecological transformation, vegetation shift

## Abstract

Under climate change, ecosystems are experiencing novel drought regimes, often in combination with stressors that reduce resilience and amplify drought’s impacts. Consequently, drought appears increasingly likely to push systems beyond important physiological and ecological thresholds, resulting in substantial changes in ecosystem characteristics persisting long after drought ends (i.e., ecological transformation). In the present article, we clarify how drought can lead to transformation across a wide variety of ecosystems including forests, woodlands, and grasslands. Specifically, we describe how climate change alters drought regimes and how this translates to impacts on plant population growth, either directly or through drought's interactions with factors such as land management, biotic interactions, and other disturbances. We emphasize how interactions among mechanisms can inhibit postdrought recovery and can shift trajectories toward alternate states. Providing a holistic picture of how drought initiates long-term change supports the development of risk assessments, predictive models, and management strategies, enhancing preparedness for a complex and growing challenge.

Earth has entered an era of rapid ecological change, a consequence of a warming climate, compounded by changes in land use and disturbance regimes (Chen et al. 2011, Millar and Stephenson 2015). Ecosystems are increasingly at risk of undergoing major and persistent shifts in community composition and function (Nolan et al. 2018), referred to as *ecological transformation* (see the glossary in box 1). Already, many communities are undergoing a gradual reorganization as species respond to shifts in climate averages (Chen et al. 2011). Alongside these gradual changes, *extreme events*, such as heatwaves, wildfires, hurricanes, and other disturbances can trigger unexpected transformations on even shorter timescales (Smith 2011, Turner et al. 2020). These pulse disturbances, especially when coupled with directional changes in climate, can push systems beyond thresholds of resilience, after which they do not return to their previous state (Harris et al. 2018). As the frequency, severity, and spatial extent of climate extremes continue to rapidly change (Ummenhofer and Meehl 2017, Seneviratne et al. 2021), many of the transformational ecological impacts of climate change can be expected to occur on the heels of single extreme events (Harris et al. 2018).

Box 1. **Glossary.**

**Drought:** an episodic deficit in water relative to average conditions

**Drought-driven ecological transformation:** Long-term, significant changes in the composition of an ecosystem that are instigated or promoted by drought, distinct from more immediate or transient drought impacts

**Compounding stressors:** in the context of drought, additional stressors or disturbances that may occur prior to, during, or following drought but are not themselves caused by drought (adapted from Paine et al. 1998, Simard et al. 2011)

**Ecological drought:** an episodic deficit in water availability that drives ecosystems beyond thresholds of vulnerability, resulting in impacts on ecosystem services (Crausbay et al. 2017)

**Ecological transformation:** a significant and persistent shift in multiple characteristics of an ecosystem, associated with a high degree of turnover in ecological communities, and which may alternatively be referred to as a state change, extreme climate event, regime shift, or vegetation type conversion (Crausbay et al. 2022)

**Ecosystem trajectory:** the pathway an ecological community takes across time, usually in terms of composition

**Evaporative demand:** the demand of water from the atmosphere (i.e., the atmosphere's drying or evaporating power, Vicente‐Serrano et al. 2020), which can be depicted by metrics such as vapor pressure deficit (VPD), the evaporative demand drought index (EDDI), or potential evapotranspiration (PET)

**Evapotranspiration:** the combined process of *evaporation* (water loss from Earth's surface directly to the air) and *transpiration* (water loss via plants)

**Extreme event:** a high intensity event that is statistically rare compared to the historical range of variability for a location, including episodes of anomalous weather/climate (e.g., droughts, floods, hurricanes) or high intensity pulse disturbances (e.g., wildfire; adapted from Harris et al. 2018, Seneviratne et al. 2021)

**Flash drought:** a drought that is characterized by an unusually rapid rate of onset or intensification, arising over a period of weeks to months (Otkin et al. 2018)

**Hydraulic failure:** severe damage to a plant's water transport system due to drought stress, resulting in desiccation and likely mortality

**Hydraulic safety margin:** the difference between the level of water stress experienced by a species under natural conditions and the level of water stress leading to hydraulic failure (Choat et al. 2012)

**Linked stressors:** in the context of drought, additional stressors or disturbances that are made more likely or more severe by drought, such as fires or pathogen outbreaks (adapted from Simard et al. 2011)

**Megadrought:** a persistent, multidecadal drought that is exceptional in severity, duration, or spatial extent (Cook et al. 2022)

**Meteorological drought:** a period of abnormally low precipitation

**Recovery:** in the context of drought, the return to predrought community composition following the end of drought

**Resilience:** in the context of drought, the ability of an ecosystem to maintain or return to predrought conditions following drought, either due to a lack of drought impact (resistance) or through rapid rates of postdrought recovery

**Snow drought:** a deficit in snowpack, either occurring from lower than normal cold season precipitation, a shift in the phase of cold season precipitation (as rain rather than snow), or from abnormally high reductions in snowpack due to melt (Mote et al. 2016)

*Drought* is a particular type of climate extreme that is increasing in frequency and severity in many regions of the world (figure 1; Seneviratne et al.^ 2021^). Although drought has shaped ecological communities and evolutionary adaptations for millennia (Rueda et al. 2017), more frequent, hotter, or novel forms of drought are now threatening established communities, even those that are considered drought adapted (Hammond et al. 2022). Moreover, the presence of additional stressors can further diminish ecological resilience, heightening the impact of even “typical” droughts (Schwalm et al. 2017, Boulton et al. 2022). Increasing drought stress, coupled with eroding resilience, can lead to impacts that are more severe than previously experienced, that are not easily reversed, and, in some cases, that result in transformation (Crausbay et al. 2020). Evidence from the paleorecord indicates that *drought-driven transformations* have previously occurred over different geologic eras, resulting in shifts in dominant vegetation that have persisted for centuries (e.g., Shuman et al. 2009, Pederson et al. 2014). For example, episodes of drought beginning around 1300 CE in Minnesota instigated a shift from oak savannas to dense forests that persisted until European settlement (Shuman et al. 2009). Contemporary examples of transformation are now appearing across multiple continents and ecosystem types, often on much more rapid timescales (figure 2; Allen and Breshears 1998, Lloret and Batllori 2021).

**Figure 1.**
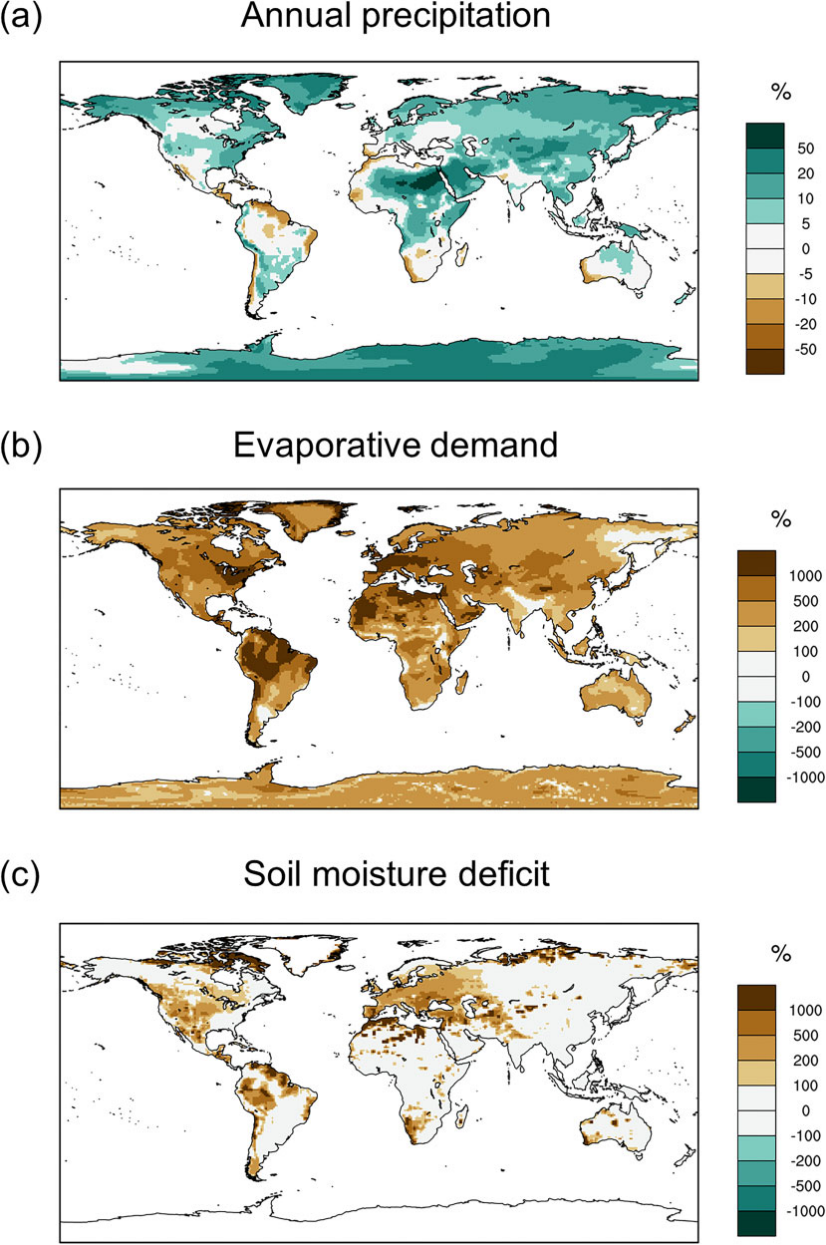
Changes in future precipitation and drought regimes. (a) Projected changes in annual precipitation by mid-century (2036–2065), indicating increased precipitation across much of the globe. (b) Projected changes in the frequency of extreme atmospheric drought events defined as monthly potential evapotranspiration values exceeding the 99th percentile threshold during a historical (1971–2000) period. (c) Projected changes in the frequency of extreme soil moisture drought events by mid-century, defined as monthly soil moisture deficits exceeding historical 99th percentile thresholds. All projection estimates are obtained from 40 simulations of the Community Earth System Model version 1 Large Ensemble (Kay et al. 2015) under the RCP8.5 emissions scenario.

**Figure 2.**
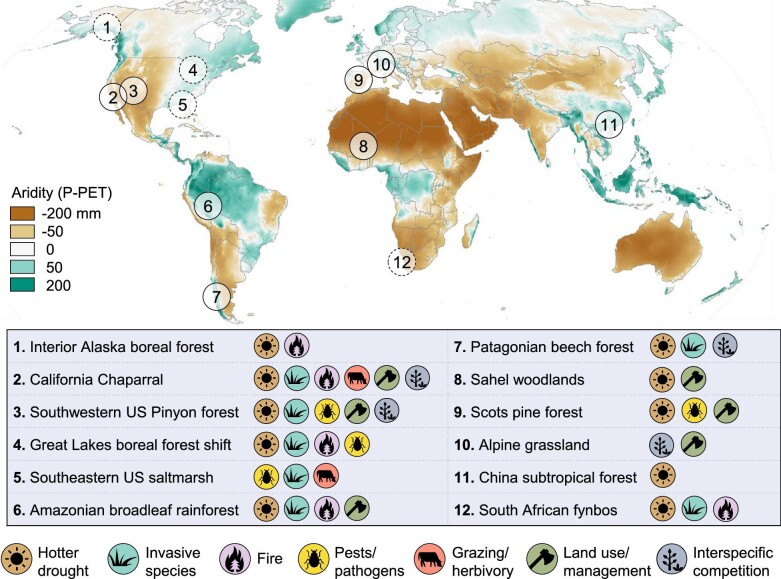
Recent examples of in-progress (solid outlines) or emerging (dotted lines) transformations that were triggered or strongly mediated by drought, compiled from literature review and previous syntheses (Martínez-Vilalta and Lloret 2016, Cobb et al. 2017, Batllori et al. 2020, Lloret and Batllori 2021). The mechanisms involved with each transformation are included where evidence exists, although the absence of a particular mechanism may reflect a lack of information. While this is not an exhaustive list, it reflects the diverse mechanisms by which drought contributes to the transformation of ecosystems across an aridity gradient. The aridity gradient was defined by the average monthly water balance (precipitation – potential evapotranspiration) over the reference period 1980–2010 using the TerraClimate data set (Abatzoglou et al. 2018). More detailed descriptions of examples are found in the supplemental material.

Increasing recognition of the potential for drought to catalyze ecological transformation (Millar and Stephenson 2015, Crausbay et al. 2020) highlights an urgent need for natural resource management to prepare for this growing challenge. Under the risk of transformation, managers may need to proactively implement interventions that increase resilience or even revisit management goals that are likely to become untenable. However, several issues currently challenge this kind of management readiness. Awareness about drought risk varies across geography and ecosystem type, with humid systems often receiving less attention in drought research and management, despite similar vulnerabilities as arid systems (Choat et al. 2012, Coble et al. 2017). Substantial uncertainty about the longer-term impacts of drought also limits management planning, especially because ecological drought research is often focused on drought's immediate, rather than long-term effects (Vilonen et al. 2022). In addition, inconsistencies across drought impact studies have hampered synthesis efforts to understand ecosystem sensitivities (Slette et al. 2019), further adding to uncertainty about ecological impacts and the risk of transformation. Finally, because drought increasingly occurs in the context of novel species assemblages and alongside other global change stressors, drought sensitivity and postdrought recovery dynamics no longer correspond to historical norms, increasing the risk of surprise (Millar and Stephenson 2015). Making decisions about how to respond to the threat of transformation requires proactive planning, but the novelty of transformations continues to defy historical experience and expectations (Crausbay et al. 2022).

In the present article, we provide a broad overview of how drought can lead to ecological transformation, highlight the relevance of this challenge across multiple ecosystems, and offer directions for future research (box 2) and planning (box 3) to better understand and prepare for this pressing phenomenon. Our emphasis is on the transformation of the dominant vegetation type in terrestrial ecosystems (including forests, woodlands, and grasslands), because this is likely to cascade to higher trophic levels and ecosystem-level processes and functions (Anderegg et al. 2013). Although other extremes can also cause transformation, a focus on drought is particularly important because the widespread distribution of increased drought stress in the coming decades (figure 1), the spatiotemporal scale of drought, and its ability to catalyze transformation across a wide range of ecosystems (figure 2) mean that it is likely to become a primary driver of ecosystem change in the twenty-first century. Our work extends and integrates previous research on drought impacts and ecological transformation by developing a generalized understanding of drought-driven transformation that applies to a broad range of ecosystems, illustrating the concept of ecological transformation through specific mechanisms and examples, and identifying and discussing potential management responses to the threat of drought-induced transformation.

## Processes involved in drought-driven transformation

**Figure 3.**
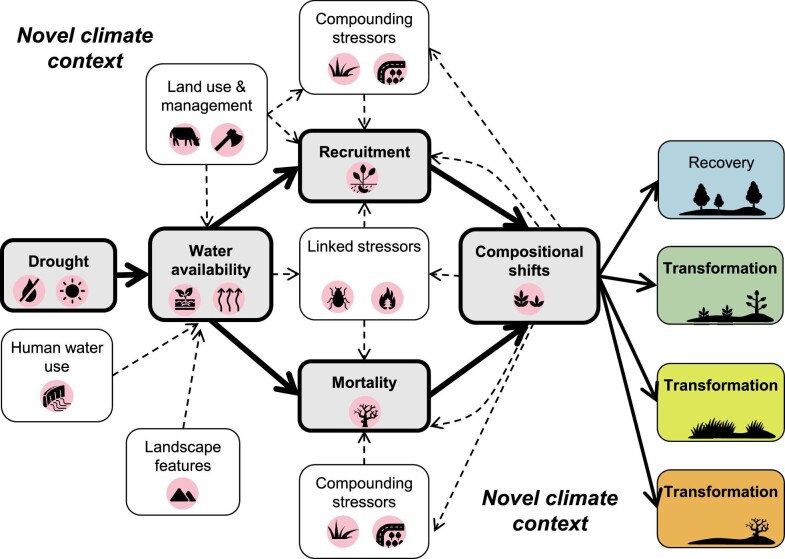
The interrelated mechanisms involved when drought triggers an ecological transformation. The solid lines represent key pathways, whereas the dotted lines are involved in some but not necessarily all transformations. The impact of drought on organisms is mediated by heterogeneity in water availability, which can be influenced by land management, human water use, and landscape features. Water deficits can alter recruitment and mortality rates, leading to shifts in community composition and structure. The effects of drought on demographic rates can be direct (i.e., because of physiological damage) or can result from drought's effects on other linked stressors (e.g., fire, insect outbreaks). Additional, compounding stressors (e.g., land-use change, invasions) may exacerbate drought's impacts or mediate postdrought dynamics in ways that promote transformation. Interactions between mechanisms (a subset of which are shown) can amplify or dampen these pathways, determining whether communities follow a trajectory that leads to recovery or to any number of trajectories that lead to ecological transformation. Novel climate conditions affect nearly all of these processes.

**Figure 4.**
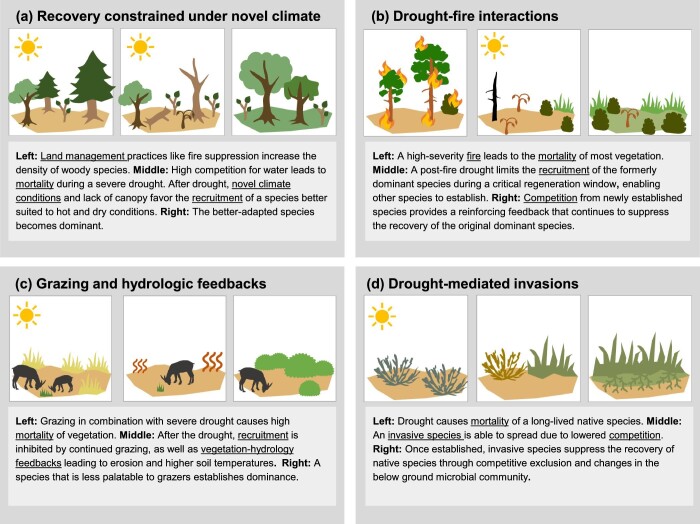
Example pathways by which drought can initiate or promote ecological transformations. These hypothetical examples are drawn from contemporary case studies but are simplified for illustrative purposes. Many pathways are possible, and we have depicted a subset to represent how interacting mechanisms can lead to transformations.

Ecological transformation has been described in a multitude of ways, including as a state change, regime shift (Scheffer et al. 2001), abrupt ecological change (Turner et al. 2020), extreme climatic event (Smith 2011), or vegetation type conversion (Jacobsen and Pratt 2018)*—*concepts that differ in their descriptions of drivers, rates of change, and feedback mechanisms (Crausbay et al. 2022). Regardless of the framework used, the key aspect of transformation that we focus on is a long-term, substantial change in the composition and characteristics of an ecosystem (e.g., a shift in the dominant vegetation type), which is difficult to reverse and requires adaptation in natural resource management (Crausbay et al. 2022). Transformations triggered by extreme events can result from changes in the driver itself (e.g., shifts in drought regimes), as well as from changes in the ecological response to that driver (e.g., ecosystem sensitivity to drought) (Smith 2011). In the following sections, we describe how both changes in drought regimes and climate context, as well as ecosystem sensitivity and recovery dynamics, contribute to an increased risk of drought-driven transformation. Importantly, drought can catalyze transformation through a variety of different pathways involving multiple and interacting mechanisms (figure 3, figure 4). Below, we review the state of the science surrounding these mechanisms and how they may be changing under twenty-first century conditions. Our focus on specific mechanisms can provide a template for conducting risk assessments and better translates the science of transformation into effective adaptation strategies and concrete management targets.

### The changing nature of drought

Although multiple factors contribute to the growing risk of ecological transformation following drought, one dominant reason is that the nature of drought is rapidly changing as a consequence of climate change, variability in teleconnection patterns (e.g., the frequency of El Niño and La Niña events), and feedbacks from land-cover change (Stark et al. 2016, Douville et al. 2021). Depending on region, sector, and impact, drought is defined and measured in a multitude of ways, including as a deficit in precipitation, unusually high atmospheric *evaporative demand*, or moisture deficits in soils and water bodies, all of which affect organismal drought stress (Redmond 2002, Slette et al. 2019). In some cases, increased drought risk results from more severe or longer-lasting deficits in precipitation (*meteorological drought*) driven by climate change, natural variability, or land–atmosphere feedbacks (Dai and Zhao 2017, Seneviratne et al. 2021). Indeed, certain regions of the world (e.g., southwestern North America, eastern Australia, western Africa) have recently experienced multidecadal precipitation deficits affecting vegetation on regional scales (Cook et al. 2020, Seneviratne et al. 2021).

However, precipitation deficits are not the primary driver of increased organismal drought stress worldwide. Precipitation is not decreasing everywhere and many locations on Earth will likely experience *higher* mean annual precipitation in the coming decades (figure 1a)—although the seasonality, precipitation form (i.e., snow versus rain), and intensity may be highly altered (Douville et al. 2021). Importantly, even in locations where annual precipitation is increasing, organismal drought stress is still predicted to worsen significantly, because of the effects of a warming climate on evaporative demand (Cook et al. 2020, Douville et al. 2021). Higher temperatures increase atmospheric evaporative demand, which, in turn, increases *evapotranspiration* rates, leading to greater soil moisture deficits (Vicente-Serrano et al. 2020) and organismal water stress (Breshears et al. 2013, Brodribb et al. 2020). Even though many plants limit transpiration in response to rising vapor pressure deficits (i.e., through stomatal closure), this also limits carbon assimilation and the functions it supports, suggesting that increasing temperatures and atmospheric demand will affect ecosystems even where they do not increase transpiration (Vicente-Serrano et al. 2020). Droughts characterized by high atmospheric evaporative demand, hotter temperatures, and the resulting low soil moisture have increased in both frequency and severity across much of the globe, and there is high confidence these types of drought will worsen in coming decades (figure 1b–1c; Cook et al. 2020, Seneviratne et al. 2021). Temperature-mediated effects on drought regimes have particular relevance because of the ubiquity of temperature increases across the globe and because the combination of high temperatures and water stress has significant impacts on plant physiology.

Temperature affects drought stress not only through rising evaporative demand but also by altering precipitation phase, snow sublimation and melt, and transpiration rates, all of which can further alter drought severity, frequency, duration (Williams et al. 2020, Wainwright et al. 2021), and seasonality (Hajek and Knapp 2022). Consequently, many regions are now experiencing forms of drought, such as *flash drought, snow drought, or megadrought* (Mote et al. 2016, Otkin et al. 2018, Cook et al. 2022), that are highly unusual within the recent historical record. Although many species are adapted to periodic water deficits, organisms may not be adapted to novel drought regimes and to unfamiliar forms of drought, which may push those species beyond their physiological limits of resilience and may lead to ecological impacts (i.e., *ecological drought*; Crausbay et al. 2020, 2022).

### Water availability

The ecological impacts of drought manifest through changes in the amount of water available to organisms and the resultant drought stress. Importantly, organismal drought stress is influenced not only by meteorological drivers (e.g., precipitation deficits and evaporative demand) but also by the human activities that modify water availability (e.g., land and water management) and the ways in which landscape features and biophysical processes (e.g., topography, organismal water use) distribute water spatially (figure 3; Cravens et al. 2023). As we move further into the twenty-first century, anthropogenic factors, such as increasing water use and changes in land use have the potential to reduce available water and exacerbate drought stress (Stark et al. 2016, Van Loon et al. 2016), meaning that even similar severities of meteorological drought may now have greater impact. For example, urbanization alters hydrology and increases evaporative demand and organismal drought sensitivity (Shields and Tague 2015, Wang et al. 2019). The expansion of nonnative species with higher water-use requirements, along with management activities that increase vegetation density (e.g., fire suppression) have both intensified competition for water, lowering per-capita water availability and exacerbating drought impacts (Cavaleri et al. 2014, Young et al. 2017).

Understanding the processes influencing water availability during drought also helps identify effective adaptation strategies. For instance, reducing ecosystem water demand can be accomplished by planting species with lower water-use requirements (Vallejo et al. 2012), and water availability can be increased by approaches such as managed aquifer recharge or soil treatments to improve infiltration (Dillon et al. 2019, Gregg and Kershner 2019). Because factors such as topography, soil type, and vegetation structure create heterogeneity in water availability, drought impacts and risk of transformation have significant spatial variability. Understanding this heterogeneity—for instance, through ecohydrological monitoring, remote sensing, hydrological modeling, or statistical techniques—is important for predicting organismal drought stress on landscape scales and for identifying refugia where managers can more easily resist drought impacts (McLaughlin et al. 2017, Cartwright et al. 2020).

### Population dynamics

Water stress may result in changes to organismal growth, survival, productivity, and reproduction (figure 3), all of which can affect ecosystem functions and services. However, transformation occurs only when drought leads to altered population growth rates among species that persist across longer time periods, ultimately leading to shifts in the dominant species (Smith 2011, Martínez-Vilalta and Lloret 2016, Esquivel‐Muelbert et al. 2019). Certain stabilizing mechanisms (e.g., reduced competition, recruitment compensation) enhance population growth and *recovery* after disturbances, promoting drought *resilience* (Lloret et al. 2012, Batllori et al. 2020). However, some of these stabilizing forces are weakening as the footprint and frequency of drought increases, and as postdrought conditions increasingly involve novel climates and biotic contexts. Demographic rates (i.e., mortality and recruitment rates) are key to understanding how communities will shift following drought and whether ecosystem trajectories are likely to lead away from recovery and toward transformation.

Drought can contribute to mortality through increased physiological stress or through indirect pathways involving additional stressors (figure 3). Direct drought-induced plant mortality is typically associated with *hydraulic failure* of the vascular system via air embolism (Adams et al. 2017, Choat et al. 2018) and involves interactions among water stress, water transport, and depletion of stored labile carbon pools, which can lead to carbon starvation (McDowell et al. 2022). A combination of high temperatures and evaporative demand strongly amplifies the risk of hydraulic failure because higher water loss via transpiration exacerbates soil moisture deficits and because plants are less able to restrict water loss under hotter temperatures (Choat et al. 2018, Brodribb et al. 2020, Marchin et al. 2022). Episodes of mass mortality are strongly associated with hotter atmospheric drought (figure 2; Breshears et al. 2013, Hammond et al. 2022), indicating that the types of drought expected to become more common in coming decades (figure 1) will heighten the risk of widespread plant mortality.

A suite of plant traits, which can help plants either avoid or tolerate water stress, shapes vulnerability to drought-induced mortality (Volaire 2018, Funk et al. 2024). Importantly, physiological drought adaptation strategies often result in a trade-off for carbon capture, leading many species of plants to operate with relatively narrow *hydraulic safety margins* to maximize carbon assimilation (Choat et al. 2012). The fact that many species, regardless of taxonomy or biome, have small safety margins suggests that, unlike other disturbances which may be geographically limited, the risk of mortality from drought is ever present across numerous ecosystems, and will be highly sensitive to the ongoing changes in drought regimes (Choat et al. 2012). Moreover, the increasing frequency of precipitation extremes and interannual variation expected under future climates may cause plants to acclimate to more mesic conditions, which could increase susceptibility to subsequent droughts (Bartlett et al. 2014, Coble et al. 2017, Jump et al. 2017).

Landscape-scale mortality events arising from drought are already leading to ecological transformations across the globe and have received widespread attention (e.g., Adams et al. 2009, Allen et al. 2015, Hammond et al. 2022). The mortality of dominant, slow-growing species such as trees is most likely to lead to rapid transformation, because the loss of reproductively mature individuals constrains recovery potential. However, mortality at any demographic stage or of other functional forms can lead to transformation if that perturbation scales up to alter relative population growth rates and species composition (Smith 2011). Even relatively small changes in background mortality rates under more frequent or longer droughts can drive gradual transformation across multiple generations if relative mortality rates shift across species (Fauset et al. 2012). These more subtle shifts may not be detected with methods designed to understand mortality at large scales (e.g., remote sensing), underscoring the importance of community-level monitoring over longer time frames (Jiao et al. 2021).

The risk of transformation is high in situations where drought-induced mortality is followed by low recruitment rates that fail to compensate for mortality (Martínez-Vilalta and Lloret 2016). Under novel climate conditions, recruitment limitation is becoming more common, particularly near species’ range edges where vegetation is in disequilibrium with current conditions (Svenning and Sandel 2013, Batllori et al. 2020). Increased temperature and evaporative demand following the loss of vegetation cover can further reduce water availability, creating dry microsite conditions that are particularly damaging to seeds and seedlings (Zellweger et al. 2020). These different postdrought conditions can quickly lead to compositional shifts, favoring a new dominant species better adapted to xeric conditions (figure 4a; Suarez and Kitzberger 2008, Batllori et al. 2020). Heightened mortality of adults, followed by postdrought recruitment failure due to seed availability, additional disturbances, or climate conditions, is an important pathway leading to rapid transformation (Martínez-Vilalta and Lloret 2016, Coop et al. 2020).

Drought-triggered transformation can also operate directly through recruitment rates. Water stress can strongly affect plant reproduction, with drought linked to lower propagule production, germination, establishment, and seedling survival in both forest and grassland systems (Pérez-Ramos et al. 2013, Zeiter et al. 2016, Harrison et al. 2018). Younger age classes are often more sensitive to drought than adults, and recruitment is a critical demographic bottleneck (Jackson et al. 2009). Therefore, drought's impacts on recruitment rates can have strong direct effects on population persistence and can lead to transformation even without a mass mortality event, especially where increased drought duration or frequency cause repeated recruitment failures across multiple years (Pozner et al. 2022). For example, lowered productivity and seedling survival during repeated droughts can deplete seedbanks in annual grasslands, inhibiting population recovery after drought ends (i.e., lowered resilience; Harrison et al. 2018). The impacts of drought on recruitment are not universally negative, however, and some species show enhanced recruitment during or following drought. This can be driven by drought-induced increases in seed dormancy or seed production*—*adaptations that may have evolved to boost recovery after disturbance in both forest and grassland species (Williamson and Ickes 2002, LaForgia et al. 2018).

As with mortality rates, differential recruitment rates among species will alter community composition and can lead to transformation, especially where differences in recruitment rates are reinforced (Stampfli and Zeiter 2004, Esquivel‐Muelbert et al. 2019). Across many systems, drought disproportionately affects dominant species, enabling the expansion of subdominant species (figure 2; Stampfli and Zeiter 2004, Fensham et al. 2015). Competition for light, nutrients, or water in the years following drought can continue to suppress the recruitment of formerly dominant species (Thrippleton et al. 2018), stabilizing compositional shifts and sometimes leading to transformation (e.g., figure 4b). For instance, in grasslands, differential recruitment among forbs, annual grasses, and perennial grasses during drought leads to shifts in composition that are amplified and reinforced by positive demographic feedbacks and competitive interactions (Stampfli and Zeiter 2004, LaForgia et al. 2018).

Species traits related to recruitment (including dispersal, seed size, drought and shade tolerance, and ability to resprout) vary widely among taxa and will shape patterns of postdrought compositional change (Batllori et al. 2020), and therefore, combining these traits with drought sensitivity traits will be key to understanding risk of transformation. Management actions that target recruitment, such as seeding and transplantation or those that enhance water availability during sensitive recruitment stages, are likely to become important in directing ecological communities toward desired states during and following drought. But, even with successful recruitment, some species may not be able to fully recover before the next drought occurs; consequently, the balance between recruitment rates and disturbance frequency will determine the likelihood of transformation (Schwalm et al. 2017). For instance, repeated droughts are already leading to transformation across Amazonia, where slow-recruiting tree species are now more likely to experience another drought during critical postdrought recovery windows (Machado‐Silva et al. 2021). Likewise, drought return intervals shape recovery probability in nonforested systems, such as grasslands (Jiao et al. 2021). An understanding of drought return intervals in relation to life histories can guide the selection of species used in restoration efforts, as well as identify locations where transformation may be more insidious, arising through reduced recruitment across repeated drought events.

### Linked and compounding stressors

Drought often occurs in combination with additional disturbances or stressors, such as fires, pathogen outbreaks, land-use conversion, or other extreme climate events. The combination of drought and other stressors, either acting on the same landscape simultaneously or occurring in sequence, can alter postdisturbance recovery trajectories and can lead to transformations that may not occur with any one stressor alone (e.g., figure 4). In some cases, additional stressors become more likely or more severe because of drought; these are referred to as *linked stressors* (figure 3). For example, drought has promoted outbreaks of insect pests and fungal pathogens, triggering mass mortality events and transformations across diverse ecosystems (figure 2) because of the relationship between water stress and defense against biotic attack (McDowell et al. 2011). Drought also decreases fuel moisture, resulting in larger fires, higher-severity fires, greater plant mortality during fires, and longer fire seasons that are more likely to cause transformation (Littell et al. 2016, Stevens-Rumann et al. 2022).

Stressors or disturbances may also occur independently of drought; *compounding stressors*, although they are not directly triggered by drought, may interact with it to drive ecological transformation (figure 3). For instance, land-use change is a globally increasing compounding stressor that can reduce ecosystem resilience to drought, such as in tropical forests, where fragmented forests are less likely to recover from drought (Boulton et al. 2022). As was previously mentioned, drought in combination with heat waves has particularly strong impacts on plant survival. Droughts occurring after other disturbances (e.g., hurricanes, wildfires) have led to delayed recovery and even transformation across multiple systems (Pratt et al. 2014, Martínez-Yrízar et al. 2018), often because drought causes recruitment failure during critical postdisturbance recovery windows (figure 4b; Davis et al. 2019, Stevens-Rumann et al. 2022). Disturbances can also drive shifts in population or community-level drought sensitivity, affecting resistance to subsequent droughts (e.g., hurricanes promote the expansion of drought vulnerable species; Smith‐Martin et al. 2022).

Biotic interactions may also act as compounding stressors, altering the effects of drought (figure 4c). For example, in savannas and grasslands, herbivory can heighten the risk of transformation by increasing plant mortality during drought, facilitating invasions during drought, and inhibiting postdrought recovery (O'Connor 1995, Loeser et al. 2007). Invasive species are also key contributors to transformations (figure 2, figure 4d, supplemental material) and affect native species’ drought sensitivity, as well as postdrought trajectories (Caldeira et al. 2015, Winkler et al. 2019). In mesic northern hardwood forests, for instance, invasive earthworms amplify drought stress for foundational tree species, contributing to the predicted transformation of temperate forests into prairies (Frelich and Reich 2010, Frelich et al. 2019).

As climate change and anthropogenic impacts alter the spatial extent, frequency, and severity of many disturbances, drought may increasingly coincide with novel stressors or extreme climate events (AghaKouchak et al. 2020) that exacerbate its impacts. Understanding where and how these stressors will interact with drought is critical to future understanding of transformation (Côté et al. 2016). For instance, identifying where drought refugia overlap with other types of refugia, such as refugia from fire, invasions, or disease, will help prioritize locations where resisting transformation is most feasible (Landesmann et al. 2015, Krawchuk et al. 2020), and reducing the impact of other stressors may be an effective strategy for mitigating drought effects.

Box 2. **High-priority research needs for understanding and predicting drought-driven transformations, along with suggestions for specific approaches that could be used to compile relevant information.**

**Figure:**
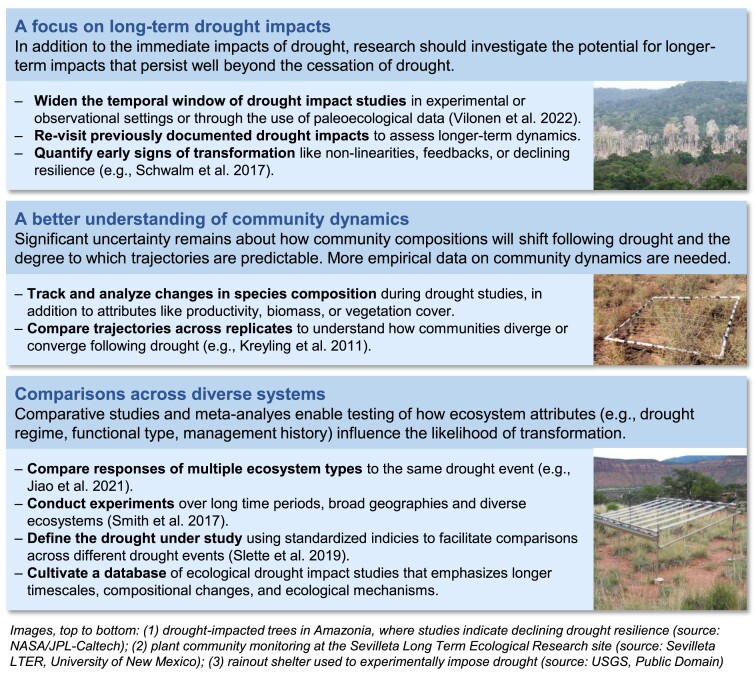

Box 3. **Emergent insights about drought-driven transformation for management preparedness.**

**Figure:**
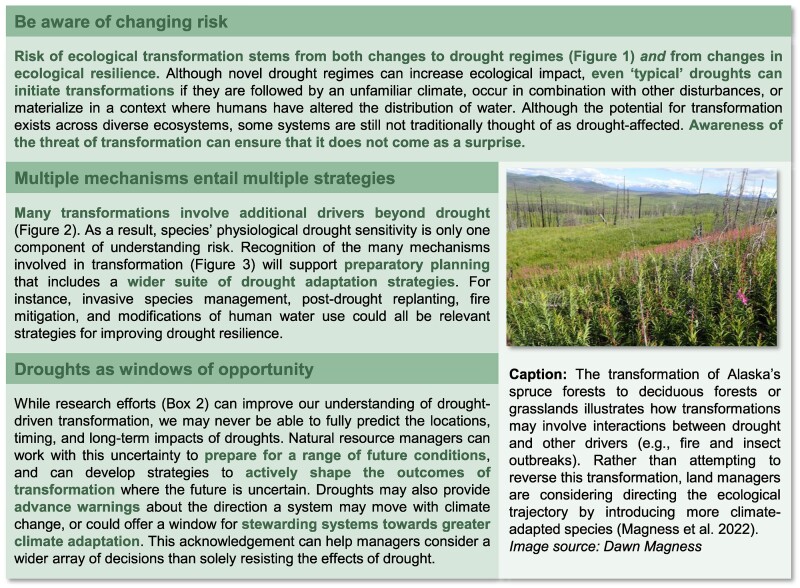

### Feedbacks

Interactions between mechanisms can amplify or dampen the effects of drought and alter the likelihood of transformation. Because recovery from disturbance can be slow and can involve transient states (Falk et al. 2022, Stevens-Rumann et al. 2022), identifying reinforcing feedback loops is key to predicting which drought impacts are likely to persist through time and ultimately lead to transformation.

Feedbacks between vegetation composition and soil properties (e.g., water content, nutrient availability, or microbial composition) often shape postdrought trajectories. For instance, numerous experimental studies in grassland communities demonstrate that drought can alter soil microbial communities, shifting the competitive dynamics among plant species (Meisner et al. 2013, Kaisermann et al. 2017). In some cases, these feedbacks between plant and microbial communities reinforce one another and create long-lasting drought impacts (Meisner et al. 2013, Kaisermann et al. 2017). However, general predictions about whether plant–soil interactions amplify or dampen the effects of drought are still elusive (van den Putten et al. 2016). Drought-induced vegetation loss may alter other soil attributes, such as increasing soil mobility, which leads to higher erosion and further amplifies vegetation loss. Intensive grazing or agricultural practices can exacerbate this; positive feedbacks among drought, land management, erosion, and vegetation loss are implicated in the transformation of the North American southern plains during the Dust Bowl (Lee and Gill 2015).

A particularly important interaction involved in many transformations is that between vegetation structure and water availability, which can reinforce alternative stable states. Compositional or structural shifts in vegetation can reduce water availability through effects on soil infiltration, solar radiation, snowmelt, and evaporation (Royer et al. 2011, Robinson et al. 2019), further affecting vegetation and reinforcing shifts in community composition (Royer et al. 2011). For example, a positive feedback between drought-induced vegetation loss and increased runoff has contributed to long-term vegetation loss in the Sahel woodlands of West Africa (figure 2, supplemental material; Trichon et al. 2018). In other cases, however, shifts in vegetation provide a dampening feedback, leading to reduced transpiration and increased water availability following drought, which enables more rapid growth (and faster recovery) for surviving individuals (Adams et al. 2012). On even larger scales, interactions between vegetation and water cycling can feed back to affect precipitation patterns and drought itself. For instance, forest loss in the Amazon basin due to drought is projected to cause major reductions in canopy transpiration and water cycling, amplifying drought conditions and transforming forests to woodlands and savanna (Wunderling et al. 2022).

### Emergent ecological trajectories

Changing population growth rates, driven by drought-induced changes to mortality and recruitment, form the basis for compositional shifts within communities. After drought, changes in population growth rates are further modified by processes such as demographic stochasticity, environmental filtering, competition or other biotic interactions, priority effects, and additional disturbances, including extreme weather events (figure 3; Anderegg et al. 2013, Young et al. 2015, Batllori et al. 2019). For instance, the order in which species establish following a drought, which can be somewhat stochastic, may shape subsequent ecosystem dynamics (i.e., priority effects), and demographic fluctuations during the initial postdrought years, when populations are small, can strongly influence community composition or even lead to local extinction (Symons and Arnott 2014, Gill et al. 2017). The trajectories of ecosystems are emergent patterns that play out over multiple spatial and temporal scales and may be defined by multiple transient states, which can be redirected by subsequent disturbances, management interventions, or stochastic processes (Cobb et al. 2017). As a result, *ecosystem trajectories* during transformation often involve a high degree of uncertainty and can lead to multiple possible futures even within the same system (Kreyling et al. 2011). In a recent analysis of forest mass-mortality events, only 20% showed self-replacement by the previously dominant species (Batllori et al. 2020), and many ecosystems exhibit multiple postdrought trajectories leading to different states (Cobb et al. 2017, Batllori et al. 2020). Notably, postdrought trajectories do not always shift communities toward more xeric species, as might be predicted (Batllori et al. 2020), highlighting that understanding species’ physiological adaptations to drought is insufficient for predicting future community composition.

Moving forward in the twenty-first century, postdrought ecological trajectories will be affected by the possibility of additional droughts or other disturbances, which can disrupt recovery (Schwalm et al. 2017), an unfamiliar climate during drought recovery windows (Crausbay et al. 2020), and an increasingly novel regional species pool (Williams and Jackson 2007). All these forces can alter trajectories away from historical patterns. Stochasticity and contingency are prominent themes in how ecological trajectories lead to transformation, with clear evidence that stochastic demographic, climate, or disturbance events can continue to reshape the trajectories of ecosystems following a drought in unpredictable ways (Kreyling et al. 2011). For example, after drought-induced die-offs of Jeffrey pines (*Pinus jeffreyi* Balf.) in Southern California, the postdrought trajectory shifted direction from oak dominated toward grass dominated with each subsequent disturbance (Safford and Vallejo 2019). This and other case studies (Cobb et al. 2017) highlight how initial ecosystem dynamics following disturbances are not necessarily indicative of future outcomes (Gill et al. 2017).

## Research and planning for drought-driven transformations

Drought-driven transformations are already occurring across the globe, rapidly in many cases, and they bring about significant and often surprising changes in ecosystems (figure 2, supplemental material). For more effective anticipation and management, a critical next step is to develop a more predictive science that assesses the risk of drought-induced transformation and its potential impacts. Several fast-growing areas of research are contributing essential tools for better anticipating the arrival or outcomes of drought in ecosystems; these include the development of indices for early ecological drought detection (Brown et al. 2008, Anderson et al. 2021), databases related to species’ drought sensitivity (Anderegg et al. 2016, Funk et al. 2024), spatially explicit assessments of drought sensitivity (Cartwright et al. 2020), and distributed experiments that quantify organismal and community-wide responses to drought across a range of geographies (Smith et al. 2017).

However, our synthesis makes it clear that unknowns regarding ecosystem responses to drought, especially on longer timescales, still limit our ability to predict and manage future transformations (box 2). Considerable uncertainty exists about which systems are most susceptible to drought-induced transformation and what postdrought trajectories will look like. Although a large body of research aims to quantify drought sensitivity and map mortality events, less common are studies that report community trajectories across longer time frames or studies that revisit severe drought impacts to understand the extent to which recovery has occurred and on what timescale. Experiments, mechanistic models, or meta-analyses that aim to quantify stochasticity and divergence in postdrought trajectories (e.g., Kreyling et al. 2011) will be key to better understanding the predictability of community assembly processes, which still represents a major gap in knowledge, especially in herbaceous systems (Wilcox et al. 2023). Comparative analyses have already generated insight into the drivers of drought-induced mortality and patterns of postdrought recovery in forest systems (Anderegg et al. 2016, Batllori et al. 2020) but are rarely applied to compare long-term drought effects across a wider range of ecosystem types. Whether broad ecosystem types differ in their resilience to drought is a key question, especially because numerous examples of transformations come from forested systems (figure 2), whereas grasslands often display a high degree of resilience (at least in terms of productivity; Wilcox et al. 2020, Xu et al. 2021). Comparative studies are needed to understand whether this pattern stems from a true difference in resilience—perhaps related to life history differences, drought adaptations, or ecosystem structure—or whether it reflects methodological bias. For example, the number of case studies of drought-induced transformation in once-forested systems may exceed those in grasslands (figure 2) because forests contain species with slower life histories (leading to longer recovery times after mortality events and, therefore, an increased likelihood of detecting transformation, real or perceived), because transformation from forested to nonforested systems is easier to measure (e.g., through remote sensing approaches), or because there is more research effort aimed at understanding forest transformation. Comparative studies could reveal how ecosystem type, along with management, drought severity, community composition, or other ecosystem attributes, influence the likelihood of transformation. Moving forward, a recognition of transformation as a distinct outcome of drought will help focus research efforts on understanding these kinds of important open questions about postdrought dynamics and transformation.

Improvements in scientific research can help narrow down the plausible outcomes of drought and guide spatial prioritization and risk assessment, but the complexity and stochasticity of ecosystems means that considerable uncertainty is likely to remain. Increasingly, research and decision-making approaches work with this inherent uncertainty to evaluate multiple plausible scenarios of future conditions, rather than a single prediction (Lawrence et al. 2021, Rangwala et al. 2021). Many frameworks now also recognize the importance of management in guiding trajectories toward alternative outcomes (Magness et al. 2022, Miller et al. 2023). Although drought adaptation strategies have primarily been focused on resisting drought impacts, the strong tendency for transformation and potential for multiple ecological trajectories can also provide opportunities to direct systems toward other desired states (box 3). For instance, postdrought trajectories could be directed toward more climate-adapted communities, and drought could present an opportunity to remove invaders or promote a particular ecosystem structure (Holmgren and Scheffer 2001, Case et al. 2019). Whether drought catalyzes preexisting trajectories toward more climate or drought-adapted communities or whether it initiates alternate, novel trajectories is a key question (box 2; Shuman et al. 2009, Griffin-Nolan et al. 2019, Batllori et al. 2020), and one that may strongly shape decisions about how and whether to intervene in drought-driven transformations. Realistically, developing an anticipatory science and management toolkit for drought-driven transformation requires recognizing the multiple mechanisms involved and integrating the growing scientific knowledge about ecological responses to drought with approaches for planning and decision-making under uncertainty.

## Supplementary Material

biae050_Supplemental_File

## Author Biography

Wynne E. Moss (wmoss@usgs.gov) was formerly a postdoctoral scientist with Conservation Science Partners, in Truckee, California, and is now a biologist with the U.S. Geological Survey, Northern Rocky Mountain Science Center, in Bozeman, Montana, both in the United States. Shelley D. Crausbay was formerly affiliated with Conservation Science Partners, in Truckee, California, at the time of this work and is now affiliated with the USDA Forest Service, in Fort Collins, Colorado, in the United States. Imtiaz Rangwala is affiliated with the North Central Climate Adaptation Science Center and with the Cooperative Institute for Research in Environmental Sciences, at the University of Colorado, in Boulder, Colorado, in the United States. Jay W. Wason is affiliated with the School of Forest Resources at the University of Maine, in Orono, Maine, in the United States. Clay Trauernicht is affiliated with the Department of Natural Resources and Environmental Management at the University of Hawai'i at Mānoa, in Honolulu, Hawai'i, in the United States. Camille S. Stevens-Rumann is affiliated with the Colorado Forest Restoration Institute in the Forest and Rangeland Stewardship Department at Colorado State University in Fort Collins, Colorado, in the United States. Anna Sala is affiliated with the Division of Biological Sciences at the University of Montana, in Missoula, Montana, in the United States. Caitlin M. Rottler is affiliated with the South Central Climate Adaptation Science Center, at the University of Oklahoma, in Norman, Oklahoma, in the United States. Gregory T. Pederson is affiliated with the U.S. Geological Survey, Northern Rocky Mountain Science Center, in Bozeman, Montana, in the United States. Brian W. Miller is affiliated with the U.S. Geological Survey, North Central Climate Adaptation Science Center, in Boulder, Colorado, in the United States. Dawn R. Magness is affiliated with the U.S. Fish and Wildlife Service, Kenai National Wildlife Refuge, in Soldotna, Alaska, in the United States. Jeremy S. Littell is affiliated with the U.S. Geological Survey, Alaska Climate Adaptation Science Center, in Anchorage, Alaska, in the United States. Lee E. Frelich is affiliated with the Department of Forest Resources at the University of Minnesota, in Saint Paul, Minnesota, in the United States. Abby G. Frazier is affiliated with the Graduate School of Geography at Clark University, in Worcester, Massachusetts, in the United States. Kimberley T. Davis was affiliated with the Department of Ecosystem and Conservation Sciences at the University of Montana at the time of this work and is currently located at the Missoula Fire Sciences Laboratory, Rocky Mountain Research Station of the USDA Forest Service, both in Missoula, Montana, in the United States. Jonathan D. Coop is affiliated with the Clark School of Environment and Sustainability, at Western Colorado University, in Gunnison, Colorado, in the United States. Jennifer M. Cartwright is affiliated with the U.S. Geological Survey, Southeast Climate Adaptation Science Center, in Raleigh, North Carolina, in the United States. Robert K. Booth is affiliated with the Earth and Environmental Science Department at Lehigh University, in Bethlehem, Pennsylvania, in the United States.
