# Correction to: Drought as an emergent driver of ecological transformation in the twenty-first century

**DOI:** 10.1093/biosci/biae085

**Published:** 2024-08-27

**Authors:** Wynne E Moss, Shelley D Crausbay, Imtiaz Rangwala, Jay W Wason, Clay Trauernicht, Camille S Stevens-Rumann, Anna Sala, Caitlin M Rottler, Gregory T Pederson, Brian W Miller, Dawn R Magness, Jeremy S Littell, Lee E Frelich, Abby G Frazier, Kimberley T Davis, Jonathan D Coop, Jennifer M Cartwright, Robert K Booth

**Affiliations:** Conservation Science Partners, Truckee, California, U.S. Geological Survey, Northern Rocky Mountain Science Center, Bozeman, Montana, United States; Conservation Science Partners, Truckee, California, USDA Forest Service, Fort Collins, Colorado, United States; North Central Climate Adaptation Science Center and Cooperative Institute for Research in Environmental Sciences, University of Colorado, Boulder, Colorado, United States; School of Forest Resources, University of Maine, Orono, Maine, United States; Department of Natural Resources and Environmental Management, University of Hawai'i, Mānoa, Honolulu, Hawai'i, United States; Colorado Forest Restoration Institute in the Forest and Rangeland Stewardship Department, Colorado State University, Fort Collins, Colorado, United States; Division of Biological Sciences, University of Montana, Missoula, Montana, United States; South Central Climate Adaptation Science Center, University of Oklahoma, Norman, Oklahoma, United States; U.S. Geological Survey, Northern Rocky Mountain Science Center, Bozeman, Montana, United States; U.S. Geological Survey, North Central Climate Adaptation Science Center, Boulder, Colorado, United States; U.S. Fish and Wildlife Service, Kenai National Wildlife Refuge, Soldotna, Alaska, United States; U.S. Geological Survey, Alaska Climate Adaptation Science Center, Anchorage, Alaska, United States; Department of Forest Resources, University of Minnesota, Saint Paul, Minnesota, United States; Graduate School of Geography, Clark University, Worcester, Massachusetts, United States; Department of Ecosystem and Conservation Sciences, University of Montana, Missoula Fire Sciences Laboratory, Rocky Mountain Research Station of the USDA Forest Service, Missoula, Montana, United States; Clark School of Environment and Sustainability, Western Colorado University, Gunnison, Colorado, United States; U.S. Geological Survey, Southeast Climate Adaptation Science Center, Raleigh, North Carolina, United States; Earth and Environmental Science Department, Lehigh University, Bethlehem, Pennsylvania, United States

This is a correction to: Wynne E Moss, Shelley D Crausbay, Imtiaz Rangwala, Jay W Wason, Clay Trauernicht, Camille S Stevens-Rumann, Anna Sala, Caitlin M Rottler, Gregory T Pederson, Brian W Miller, Dawn R Magness, Jeremy S Littell, Lee E Frelich, Abby G Frazier, Kimberley T Davis, Jonathan D Coop, Jennifer M Cartwright, Robert K Booth, Drought as an emergent driver of ecological transformation in the twenty-first century, *BioScience*, 2024; https://doi.org/10.1093/biosci/biae050

In the originally published version of this manuscript, the Green and Blue figures, labelled box 2 and 3 were erroneously swapped.

The Blue figure should be in Box 2 and accompanied by the title: ***High-priority research needs for understanding and predicting drought-driven transformations, along with suggestions for specific approaches that could be used to compile relevant information.***

The Green figure should be in Box 3 and accompanied by the title: ***Emergent insights about drought-driven transformation for management preparedness.***

Emendation has been made in the article.

