# Supplementary material for: Drought as an emergent driver of ecological transformation in the twenty-first century
Source: Bioscience. 2024 Jul 10;74(8):524–38. doi: 10.1093/biosci/biae050 (PMC11770345; doi:10.1093/biosci/biae050)
Supplement: biae050_Supplemental_File [file biae050_supplemental_file.docx]

Supplementary Materials for:

Moss WE, Crausbay SD, Rangwala I, Wason JW, Trauernicht C, Stevens-Rumann CS, Sala A, Rottler CM, Pederson GT, Miller BW, Magness DR, Littell JS, Frelich LE, Frazier AG, Davis KT, Coop JD, Cartwright JM, Booth RK. *Drought as an emergent driver of ecological transformation in the 21^st^ century.*

**APPENDIX A: CASE STUDIES**

We compiled examples of recent (1950s – present) transformational ecological droughts (TEDs) from the literature. Many case studies were obtained from previous reviews and meta-analyses (Martínez-Vilalta and Lloret 2016, Cobb et al. 2017, Ratajczak and Ladwig 2019, Batllori et al. 2020, Lloret and Batllori 2021) and were supplemented with other examples. To be included herein, we required case studies to (1) document a compositional shift in vegetation communities that was attributed to a drought or series of droughts and (2) indicate that recovery was unlikely. Many case studies clearly demonstrated a lack of recovery in the years following drought or showed that shifts in composition were stable across time, and we considered these to be clear examples of TEDs. Other examples of TEDs showed compositional shifts, but not enough time had passed to clearly define the shift as persistent. In these cases, authors often inferred the stability based upon plant or ecosystem traits, early evidence of regeneration failure, or modeling approaches. We include these as ‘emerging’ examples of TEDs. We note that this is not an exhaustive list but is presented to demonstrate that TEDs are occurring across many geographies and ecosystems, and that multiple different mechanisms are involved. Additional descriptions of each example are provided below.

1. **Interior Alaska boreal forest (emerging)**

**Location:** Interior Alaska (United States)

**Transformation:** spruce forest to deciduous forest or grassland

**Description:** Spruce forests (dominated by black spruce [*Picea mariana*] or white spruce [*Picea glauca*]) in the interior boreal forest of Alaska are at risk of transitioning to aspen forest (*Populus tremuloides*) or other forms of temperate deciduous forest, and in some cases, to grassland. This is consistent with shifts in the overall distribution of the boreal forest range, which are predicted by vegetation modeling (Lucht et al. 2006). Spruce decline is likely driven by the interaction of drought and more frequent fires, such that post-fire recovery is increasingly limited by lack of moisture (Mann et al. 2012, Baltzer et al. 2021). Declines of spruce occur even in unburned forests, suggesting warmer temperatures and drought stress limit adult growth and survival in addition to suppressing post-fire regeneration (Barber et al. 2000, Beck et al. 2011). Evaporative demand in Alaska has increased in recent decades (Beck et al. 2011), which is reducing growth and survival of numerous boreal species. Forest disturbance modeling (Mann et al. 2012) suggests that spruce will be replaced by early successional deciduous forest, while other studies suggest that multiple trajectories are possible, with some spruce forests transitioning to deciduous forest, and others being replaced by non-forest types (Baltzer et al. 2021). Because the shifts in biome are relatively recent (since the mid-1990s; Beck et al. 2011) and are still ongoing, we have characterized this TED as ‘emerging’.

1. **California chaparral**

**Location:** southern California (United States)

**Transformation:** closed-canopy chaparral to open-canopy chaparral or grassland

**Description:** Drought, including the exceptional ones of 2002 – 2003 and 2012 – 2015, is a major factor in the transformation of chaparral ecosystems in southern California, which have converted from closed-canopy shrublands into open canopy systems dominated by different shrub species or by (often non-native) herbaceous species (Park and Jenerette 2019, Pratt 2022). There are several pathways by which drought can catalyze a permanent change in chaparral vegetation (Jacobsen and Pratt 2018). In one pathway, drought triggers a mass mortality event in mature chaparral shrubs (Coates et al. 2015, Venturas et al. 2016), as has been observed during the recent hotter droughts in California (Diffenbaugh et al. 2015, Venturas et al. 2016). Recruitment can then be limited by the physical conditions of an open canopy as well as competition with non-native annuals, which can better establish without canopy cover (Park et al. 2018, Park and Jenerette 2019). Land use legacies involving habitat conversion, grazing, and changes to the fire regime have previously promoted the colonization of invasive grasses, making this shift more likely (Park and Jenerette 2019). Other pathways to transformation in chaparral systems involve drought limiting post-fire regeneration or facilitating more frequent fires (Pratt et al. 2014, Jacobsen and Pratt 2018). Feedbacks with soil chemistry, soil moisture profiles, and microbial community composition can reinforce compositional shifts (Dickens and Allen 2014, Park and Jenerette 2019). Recruitment and mortality rates to both fire and drought are strongly shaped by traits like re-seeding ability and root depth, and the trajectory of change is sensitive to the particular sequence of drought and fire (Jacobsen and Pratt 2018). As a result of the complex mechanisms involved, multiple trajectories are possible in chaparral systems, including a shift in the type of dominant woody species, or conversion to herbaceous cover (Jacobsen and Pratt 2018).

1. **Southwestern US pinyon forest**

**Location:** Arizona, New Mexico, Colorado, and Utah (United States)

**Transformation:** pinyon pine-dominated woodlands to juniper-dominated woodlands or herbaceous cover

**Description**: Hotter, multi-year droughts between 1996 and 2005 has led to the mass mortality of pinyon pines (*Pinus edulis*) across the southwestern United States, which are either the dominant or co-dominant species across much of the region (Breshears et al. 2005, Floyd et al. 2009). Drought is linked to increased susceptibility to bark beetle (*Ips confusus*) attack, which is the proximate cause of mortality (Breshears et al. 2005, 2009). Preceding wet years, along with fire suppression led to increased stand density and competition over ecologically available water, likely increasing mortality rates during drought (Breshears et al. 2005). Compared to pinyon pine, junipers (*Juniperus monosperma*) have higher survival rates and higher regeneration rates after drought, which has shifted community composition towards juniper dominance in many locations across the southwest (Mueller et al. 2005, Floyd et al. 2015, Redmond et al. 2015, 2018). In other locations, die-off of both pinyon and juniper has led to increased cheatgrass (*Bromus tectorum*) cover or dominance by understory herbaceous species (Kane et al. 2011, Redmond et al. 2015). The loss of mature nurse trees, lack of seed source, and competition with expanding herbaceous ground cover has continued to suppress pinyon pine regeneration for more than a decade after drought, reinforcing the transformation (Mueller et al. 2005, Floyd et al. 2015, Redmond et al. 2015, 2018).

1. **Great Lakes boreal forest shift (emerging)**

**Location:** Great Lakes region including Michigan, Wisconsin, Minnesota, and Ontario (United States and Canada)

**Transformation:** southern boreal forest to temperate forest or grasslands

**Description:** Drought is expected to play a strong role in the transformation of boreal forests near their southern range edge (Frelich and Reich 2010). While southern boreal forests are gradually declining over generations due to a warming climate, drought can greatly speed the rate of transformation by triggering die-offs of mature boreal trees (Frelich et al. 2021). The risk of drought-induced die-offs is growing in the Great Lakes region as hotter and more frequent droughts during the summer months are expected (Frelich and Reich 2010). Moreover, invasions by non-native earthworms (*Lumbricus terrestris, Lumbricus rubellus,* and *Aporrectodea* spp.) have increased ecosystem susceptibility to drought by altering soil moisture content (Frelich et al. 2019). Following overstory mortality, temperate understory species that are more well-adapted to a warmer and drier climate are expected to regenerate at greater rates, shifting forest composition (Frelich et al. 2021). At the same time, other increasingly frequent stressors like windthrow, herbivory, pathogens, and fire are suppressing boreal tree regeneration and furthering transformation (Frelich and Reich 2010). As a result, boreal forest currently dominated by jack pine (*Pinus banksiana*), black spruce (*Picea mariana*), balsam fir (*Abies balsamea*), aspen (*Populus tremuloides*) and paper birch (*Betula papyrifera*) could convert to temperate forest dominated by more xeric species like oaks (*Quercus* spp; Gustafson and Sturtevant 2013, Frelich et al. 2021). Depending on location, transformation into savanna or grasslands is also possible (Toot et al. 2020, Frelich et al. 2021). A shift in the boreal forest range due to drought is anticipated based upon current rates of climatic change, early evidence of forest shifts, ecological theory, and vegetation modeling (Frelich and Reich 2010, Gustafson and Sturtevant 2013, Frelich et al. 2021). However, recent research still categorizes this as an ‘emerging’ transformation that has not yet occurred on larger scales (Frelich et al. 2021).

1. **Southeastern US saltmarsh (emerging)**

**Location:** Atlantic and Gulf coast locations in Georgia, Louisiana, and South Carolina (United States)

**Transformation:** cordgrass-dominated salt marshes to mudflats or open water

**Description**: Droughts have triggered die-offs of the dominant salt marsh vegetation (cordgrass, *Spartina alterniflora*) across multiple locations in the southeastern United States, leading to conversion into mudflats or open water (Alber et al. 2008). During droughts in the late 1990s and early 2000s (e.g., 1990 –1995 [Florida Panhandle], 1999 [Louisiana], 2001–2002 [Georgia and South Carolina]) , reduced precipitation led to lower freshwater flows into marshes, which altered soil water content and chemistry and resulted in high cordgrass mortality (McKee et al. 2004, Alber et al. 2008, Li et al. 2022). Cordgrass mortality during drought is further amplified by high rates of herbivory, as snails and crabs concentrate on remaining patches of cordgrass (Silliman et al. 2005, Angelini et al. 2018). Drought resilience is strongly related to the presence of a mutualist species (the ribbed mussel *Geukensia demissa*), which promotes cordgrass survival and recovery through a variety of mechanisms (Bertness 1984, Angelini et al. 2016). Loss of cordgrass reduces mussel densities, creating a positive feedback that inhibits recovery (Bertness et al. 2015). Moreover, mussel populations are threatened by feral hogs, which are spreading throughout the southeast; thus, the ability of mussels to buffer against drought may be declining (Hensel et al. 2021). A feedback between vegetation loss and erosion can further reduce recovery rates of cordgrass (Alber et al. 2008). Depending on the rates of recovery, salt marshes may either fully recover, undergo compositional shifts to more drought resistant vegetation, or transform into de-vegetated mudflats or open water (McKee et al. 2004, Alber et al. 2008, Marsh et al. 2016). Although recovery has been deemed unlikely in many locations due to the aforementioned feedbacks and stabilizing mechanisms (McKee et al. 2004, Rolando et al. 2023), many studies have focused on detecting dieback rather than tracking longer term recovery dynamics; therefore we categorized this transformation as ‘emerging’(Rolando et al. 2023).

1. **Amazonian broadleaf rainforest**

**Location:** Amazon basin

**Transformation:** moist tropical forests to dry forests or savannas

**Description:** Large areas of the Amazon basin’s moist tropical forests have been lost since the early 2000s, and drought is thought to bring Amazonian forests closer to a crucial tipping point (Boulton et al. 2022). Recent severe and hot droughts in 2005, 2007, and 2010 have caused major declines in forest biomass due to moisture stress (Phillips et al. 2009) and have also led to widespread and high intensity fires (Brando et al. 2014). Burned forests can be replaced by invasive grasses, which can trigger a positive grass-fire feedback and an abrupt ecosystem transformation towards an open canopy savanna ecosystem (Brando et al. 2014). Loss of canopy cover also decreases atmospheric moisture recycling and exacerbates drying, reinforcing the effects of drought (Wunderling et al. 2022). Land use change amplifies these processes (Boulton et al. 2022), as deforestation can increase land surface temperatures and fuel loads (Brando et al. 2014). Amazon forest composition is also shifting without the influence of fires, albeit more slowly. As droughts have increased in duration and frequency, relative mortality and recruitment rates among species have shifted, favoring the dominance of xeric species (Esquivel‐Muelbert et al. 2019). Although many studies agree that Amazon moist tropical forests will decline due to increasing drought, multiple future states (e.g., seasonal forests, dry forests, or savannas) are possible, depending on moisture and disturbance regimes (Malhi et al. 2009, Bonal et al. 2016, Esquivel‐Muelbert et al. 2019).

1. **Patagonian beech forest**

**Location:** northern Patagonia, Argentina

**Transformation:** beech-dominated forest to beech-cedar co-dominated forests

**Description:** An evergreen forest in northern Patagonia, Argentina, dominated by beech (*Nothofagus dombeyi*) individuals experienced a severe and hot drought from 1998–1999 (Suarez and Kitzberger 2008). This resulted in mortality of both adult and sapling beech individuals, reducing their density by more than 50% in some areas (Suarez and Kitzberger 2008). A sub-canopy species, the Chilean cedar (*Austrocedrus chilensis*) was more drought-tolerant, experiencing very little mortality; this difference in mortality rates shifted composition towards more equal dominance by the two species (Suarez and Kitzberger 2008) . Moreover, unlike treefall gaps, which maintain an equilibrium between the two species, gaps created by drought strongly favored recruitment pulses of Chilean cedar and understory shrubs, which are more shade and drought tolerant (Suarez and Kitzberger 2008, 2010). Several factors, including changes in the forest microenvironment, lack of seed source, and the presence of invasive shrubs in the understory, have continued to suppress the recruitment of beech saplings, and thus reinforce the compositional shifts (Suarez and Kitzberger 2008, Suarez and Sasal 2012). Repeated droughts could further alter this system’s trajectory, leading to even more overstory mortality and a greater compositional shift which would favor dominance by resprouting shrubs and grasses (Suarez and Kitzberger 2010, Cobb et al. 2017).

1. **Sahel woodlands**

**Location**: west African Sahel region (Mali, Senegal)

**Transformation**: loss of woody cover and replacement by more xeric or herbaceous species, or shift to de-vegetated state

**Description:** From the late 1960s to 1990s, the Sahel region experienced a period of extremely low rainfall, during which several distinct drought events occurred (Lebel and Ali 2009). Precipitation deficits were also associated with warmer temperatures which exacerbated plant stress (Gonzalez et al. 2012) and resulted in severe declines in the biodiversity and density of woody species (e.g., *Pterocarpus lucens, Combretum micranthum*) at regional scales (Gonzalez et al. 2012, Trichon et al. 2018). The loss of woody cover altered hydrology from run-on to run-off, with erosion forming gullies that redirected water away from remaining vegetation (Trichon et al. 2018). This positive vegetation-hydrology feedback reinforced the decline of woody vegetation (Trichon et al. 2018, Wendling et al. 2019) and continued to suppress regeneration even when rains returned. Anthropogenic pressures (e.g., livestock browsing and wood collection) also likely contributed to drought vulnerability by reducing vegetation cover and increasing albedo (Gonzalez 2001). Since the return of wetter conditions, vegetation recovery has been observed in some locations, although recovery trajectories differ across the Sahel, likely due to differences in climate regime, land use, and soil properties (Hiernaux et al. 2009). In some locations, woody species (e.g., *Acacia* spp.) are regenerating ((Hiernaux et al. 2009), although they are often different than the formerly dominant species and represent a shift towards more xeric communities (Hiernaux et al. 2009, Trichon et al. 2018). Increases in herbaceous vegetation have been observed across much of the Sahel (i.e., the ‘Sahel greening’, Dardel et al. 2014). Yet, it is clear that large areas of woody vegetation have still not recovered decades after the major droughts, and due to the change in hydrology, this appears to be a permanent shift in ecosystem type across much of the Sahel (Gonzalez 2001, Trichon et al. 2018).

1. **Scots pine forest**

**Location:** central and southern Europe, including Spain, Switzerland, and Germany

**Transformation:** Scots pine-dominated forests to oak- or juniper- dominated forests

**Description:** A number of recent droughts (e.g., 1956 – 1974, 1994 – 1995, 2005, 2012) are altering the composition of Mediterranean forests in central and southern Europe, which have historically been dominated by Scots pine (*Pinus sylvestris*). Loss of Scots pine canopy cover has been observed in numerous locations across Europe, usually following drought events (Bigler et al. 2006, Vilà-Cabrera et al. 2011, Rigling et al. 2013, Gazol et al. 2018a), and the southern range edge appears at high risk of local extinction (Camarero et al. 2015, Gazol et al. 2018b). Droughts reduce Scots pine growth, induce defoliation, and can result in mass mortality events, with warmer temperatures exacerbating these processes (Bigler et al. 2006, Camarero et al. 2015, Haberstroh et al. 2022). Drought stress is also associated with fungal infections, which can further reduce water transport and predispose trees to mortality (Aguadé et al. 2015, Camarero et al. 2015). Land management legacies also shape patterns of drought mortality. Abandonment of logging has led to high stand densities in some locations, increasing competition for ecologically available water and leading to higher mortality in stands that are now unmanaged (Vilà-Cabrera et al. 2013). After drought, Scots pine regeneration is often too low to enable recovery, particularly in dry sites (Vilà-Cabrera et al. 2011, 2013, Galiano et al. 2013, Gazol et al. 2018a). Low rates of pine recruitment may be driven by reduced seed availability and low post-drought seedling survival under a hotter and drier climate (Galiano et al. 2010, Vilà-Cabrera et al. 2013, Gazol et al. 2018a). Meanwhile, previously sub-dominant species, like oaks (*Quercus* spp., Galiano et al. 2013, Rigling et al. 2013, Vilà-Cabrera et al. 2013, Gazol et al. 2018) and juniper (*Juniperus thurifera*; Gazol et al. 2018a), which are more drought-tolerant, demonstrate high recruitment rates after drought. Shade-tolerant oak seedlings in the understory are able to gain a competitive advantage after drought and have enhanced growth (Galiano et al. 2013). Thus, a shift towards oak (and perhaps juniper) dominance is occurring across much of central and southern Europe.

1. **Alpine grassland**

**Location:** southern Alps, Switzerland

**Transformation:** decrease in dominant grasses and increase in sub-dominant forbs

**Description:** Three successive years of exceptional summer drought (1989 – 1991) led to a persistent compositional shift in a semi-natural perennial grassland in the southern Alps that was managed for haymaking (Stampfli and Zeiter 2004). Although both grasses and forbs appeared to decline similarly during the drought, in the years following drought, differential recruitment rates led to a decline in the dominant grass species (e.g., *Bromus erectus, Brachypodium pinnatum*) and increases in rarer species of forbs (e.g., *Plantago lanceolata, Scabiosa columbaria*). Competitive release due to a resource pulse after the drought is a likely mechanism (Stampfli and Zeiter 2004, 2008). Traits related to germination also favored forbs over grasses, as forbs had more abundant seed sources and germinated earlier in the year during favorable conditions (Stampfli and Zeiter 2004, 2008) . The compositional shift was stabilized during the following wet years, when established species were able to expand, thus changes to grassland communities are likely to persist (Stampfli and Zeiter 2004) . Land management practices could strongly shape trajectories, as the timing of harvest favors plants with certain germination phenologies (Stampfli and Zeiter 2008).

1. **China subtropical forest**

**Location:** Guandong Province, China

**Transformation**: mixed tropical forest dominated by mesic species to mixed tropical forest with smaller xeric species and shrubs

**Description:** Subtropical evergreen forests in southern China have undergone compositional shifts across the past three decades (Zhou et al. 2013, 2014, Li et al. 2015). Several lines of evidence indicate that these shifts are associated with an increasing frequency of droughts, especially between 1990 and 2010. The number of rain-free days in this region has increased across time, as have dry-season temperatures, although mean annual precipitation has not strongly changed (Zhou et al. 2011, 2013, Li et al. 2015). Moreover, temporal trends in composition correlate highly with trends in drought variables (Zhou et al. 2013, 2014). Specifically, compositional changes are associated with decreases in soil moisture during the dry season, combined with high VPD (Zhou et al. 2013). Changes in the drought regime have favored the survival and recruitment of more drought-adapted species. Larger tree species have decreased while those with rapid growth rates and high drought tolerance (e.g., high hydraulic conductivity and turgor loss point) have increased (Li et al. 2015). Overall, forest composition and structure are shifting towards denser forests with smaller trees and shrubs (Zhou et al. 2013, 2014). This case study is somewhat unusual in the absence of additional disturbances or anthropogenic factors, suggesting that droughts alone could drive transformation, especially if they are repeated across time and disproportionately favor the population growth of certain species (Zhou et al. 2014, Li et al. 2015).

1. **South African fynbos (emerging)**

**Location:** Cape Floristic Region, South Africa

**Transformation:** declines in graminoids, herbs, and post-fire resprouters

**Description:** More than four decades of data show a reorganization of vegetation in the Fynbos biome of South Africa due to interactions between drought and fire. The Fynbos ecosystem is fire-adapted, with fires typically occurring a few times a century, and plants in this region have evolved numerous strategies for post-fire recovery (Keeley et al. 2011). Recruitment during the first year after fire represents an important bottleneck, and both germination and seedling survival are sensitive to drought conditions during this critical window (Le Maitre and Midgley 1992, Mustart et al. 2012). The drought regime in the region is changing, with increasing temperatures and more consecutive hot and dry days (van Wilgen et al. 2016, Slingsby et al. 2017). Therefore, it has become increasingly likely that the post-fire regeneration window overlaps with a drought event (Slingsby et al. 2017). This has led to a decline in species diversity across the past 40 years, with the largest declines in re-sprouting species, graminoids and herbs (Slingsby et al. 2017) . Because these species are typically among the first to recover after fire, their sensitivity to drought means that postfire vegetation recovery is being slowed (Slingsby et al. 2017) . Re-sprouting species have lower rates of establishment from seed, and are highly sensitive to adult mortality, which could explain their declines. On the other hand, shrubs, particularly those with deeper root systems are more resilient, and overall, the community has shifted towards more drought-adapted species with higher temperature tolerances (Slingsby et al. 2017) . The introduction of invasive woody species may have also contributed to transformation, by reducing soil moisture, competing with native vegetation, and reducing the seedbank of native species (Le Maitre et al. 2011), all of which would erode resilience to drought and fires (Slingsby et al. 2017) . While this pattern has only recently emerged, the changes in drought regimes are likely to continue to drive changes in Fynbos diversity and composition.

**References**

Aguadé D, Poyatos R, Gómez M, Oliva J, Martínez-Vilalta J. 2015. The role of defoliation and root rot pathogen infection in driving the mode of drought-related physiological decline in Scots pine (*Pinus sylvestris*). Tree physiology 35: 229–242.

Alber M, Swenson EM, Adamowicz SC, Mendelssohn IA. 2008. Salt marsh dieback: an overview of recent events in the US. Estuarine, Coastal and Shelf Science 80: 1–11.

Angelini C, Griffin JN, van de Koppel J, Lamers LP, Smolders AJ, Derksen-Hooijberg M, van der Heide T, Silliman BR. 2016. A keystone mutualism underpins resilience of a coastal ecosystem to drought. Nature Communications 7: 12473.

Angelini C, van Montfrans SG, Hensel MJ, He Q, Silliman BR. 2018. The importance of an underestimated grazer under climate change: how crab density, consumer competition, and physical stress affect salt marsh resilience. Oecologia 187: 205–217.

Baltzer JL, Day NJ, Walker XJ, Greene D, Mack MC, Alexander HD, Arseneault D, Barnes J, Bergeron Y, Boucher Y. 2021. Increasing fire and the decline of fire adapted black spruce in the boreal forest. Proceedings of the National Academy of Sciences 118: e2024872118.

Batllori E, Lloret F, Aakala T, Anderegg WR, Aynekulu E, Bendixsen DP, Bentouati A, Bigler C, Burk CJ, Camarero JJ. 2020. Forest and woodland replacement patterns following drought-related mortality. Proceedings of the National Academy of Sciences 117: 29720–29729.

Beck PS, Juday GP, Alix C, Barber VA, Winslow SE, Sousa EE, Heiser P, Herriges JD, Goetz SJ. 2011. Changes in forest productivity across Alaska consistent with biome shift. Ecology letters 14: 373–379.

Bertness MD. 1984. Ribbed mussels and Spartina alterniflora production in a New England salt marsh. Ecology 65: 1794–1807.

Bertness MD, Brisson CP, Crotty SM. 2015. Indirect human impacts turn off reciprocal feedbacks and decrease ecosystem resilience. Oecologia 178: 231–237.

Bigler C, Bräker OU, Bugmann H, Dobbertin M, Rigling A. 2006. Drought as an inciting mortality factor in Scots pine stands of the Valais, Switzerland. Ecosystems 9: 330–343.

Bonal D, Burban B, Stahl C, Wagner F, Hérault B. 2016. The response of tropical rainforests to drought—lessons from recent research and future prospects. Annals of Forest Science 73: 27–44.

Boulton CA, Lenton TM, Boers N. 2022. Pronounced loss of Amazon rainforest resilience since the early 2000s. Nature Climate Change 12: 271–278.

Brando PM, Balch JK, Nepstad DC, Morton DC, Putz FE, Coe MT, Silvério D, Macedo MN, Davidson EA, Nóbrega CC. 2014. Abrupt increases in Amazonian tree mortality due to drought–fire interactions. Proceedings of the National Academy of Sciences 111: 6347–6352.

Breshears DD, Cobb NS, Rich PM, Price KP, Allen CD, Balice RG, Romme WH, Kastens JH, Floyd ML, Belnap J. 2005. Regional vegetation die-off in response to global-change-type drought. Proceedings of the National Academy of Sciences 102: 15144–15148.

Breshears DD, Myers OB, Meyer CW, Barnes FJ, Zou CB, Allen CD, McDowell NG, Pockman WT. 2009. Tree die‐off in response to global change‐type drought: mortality insights from a decade of plant water potential measurements. Frontiers in Ecology and the Environment 7: 185–189.

Camarero JJ, Gazol A, Sangüesa‐Barreda G, Oliva J, Vicente‐Serrano SM. 2015. To die or not to die: early warnings of tree dieback in response to a severe drought. Journal of Ecology 103: 44–57.

Coates AR, Dennison PE, Roberts DA, Roth KL. 2015. Monitoring the impacts of severe drought on southern California chaparral species using hyperspectral and thermal infrared imagery. Remote Sensing 7: 14276–14291.

Cobb RC, Ruthrof KX, Breshears DD, Lloret F, Aakala T, Adams HD, Anderegg WR, Ewers BE, Galiano L, Grünzweig JM. 2017. Ecosystem dynamics and management after forest die‐off: A global synthesis with conceptual state‐and‐transition models. Ecosphere 8: e02034.

Dardel C, Kergoat L, Hiernaux P, Mougin E, Grippa M, Tucker C. 2014. Re-greening Sahel: 30 years of remote sensing data and field observations (Mali, Niger). Remote Sensing of Environment 140: 350–364.

Dickens S, Allen E. 2014. Exotic plant invasion alters chaparral ecosystem resistance and resilience pre-and post-wildfire. Biological Invasions 16: 1119–1130.

Diffenbaugh NS, Swain DL, Touma D. 2015. Anthropogenic warming has increased drought risk in California. Proceedings of the National Academy of Sciences 112: 3931–3936.

Esquivel‐Muelbert A, Baker TR, Dexter KG, Lewis SL, Brienen RJ, Feldpausch TR, Lloyd J, Monteagudo‐Mendoza A, Arroyo L, Álvarez-Dávila E. 2019. Compositional response of Amazon forests to climate change. Global Change Biology 25: 39–56.

Floyd ML, Clifford M, Cobb NS, Hanna D, Delph R, Ford P, Turner D. 2009. Relationship of stand characteristics to drought‐induced mortality in three Southwestern piñon–juniper woodlands. Ecological Applications 19: 1223–1230.

Floyd ML, Romme WH, Rocca ME, Hanna DP, Hanna DD. 2015. Structural and regenerative changes in old-growth piñon–juniper woodlands following drought-induced mortality. Forest Ecology and Management 341: 18–29.

Frelich LE, Blossey B, Cameron EK, Dávalos A, Eisenhauer N, Fahey T, Ferlian O, Groffman PM, Larson E, Loss SR. 2019. Side‐swiped: ecological cascades emanating from earthworm invasions. Frontiers in Ecology and the Environment 17: 502–510.

Frelich LE, Montgomery RA, Reich PB. 2021. Seven ways a warming climate can kill the southern boreal forest. Forests 12: 560.

Frelich LE, Reich PB. 2010. Will environmental changes reinforce the impact of global warming on the prairie–forest border of central North America? Frontiers in Ecology and the Environment 8: 371–378.

Galiano L, Martínez‐Vilalta J, Eugenio M, Granzow‐de la Cerda Í, Lloret F. 2013. Seedling emergence and growth of *Quercus* spp. following severe drought effects on a *Pinus sylvestris* canopy. Journal of Vegetation Science 24: 580–588.

Galiano L, Martínez-Vilalta J, Lloret F. 2010. Drought-induced multifactor decline of Scots pine in the Pyrenees and potential vegetation change by the expansion of co-occurring oak species. Ecosystems 13: 978–991.

Gazol A, Camarero JJ, Sangüesa-Barreda G, Vicente-Serrano SM. 2018a. Post-drought resilience after forest die-off: shifts in regeneration, composition, growth and productivity. Frontiers in Plant Science 9: 1546.

Gazol A, Camarero JJ, Vicente‐Serrano SM, Sánchez‐Salguero R, Gutiérrez E, de Luis M, Sangüesa‐Barreda G, Novak K, Rozas V, Tíscar PA. 2018b. Forest resilience to drought varies across biomes. Global Change Biology 24: 2143–2158.

Gonzalez P. 2001. Desertification and a shift of forest species in the West African Sahel. Climate Research 17: 217–228.

Gonzalez P, Tucker CJ, Sy H. 2012. Tree density and species decline in the African Sahel attributable to climate. Journal of Arid Environments 78: 55–64.

Gustafson EJ, Sturtevant BR. 2013. Modeling forest mortality caused by drought stress: implications for climate change. Ecosystems 16: 60–74.

Haberstroh S, Werner C, Grün M, Kreuzwieser J, Seifert T, Schindler D, Christen A. 2022. Central European 2018 hot drought shifts scots pine forest to its tipping point. Plant Biology 24: 1186–1197.

Hensel MJ, Silliman BR, van de Koppel J, Hensel E, Sharp SJ, Crotty SM, Byrnes JE. 2021. A large invasive consumer reduces coastal ecosystem resilience by disabling positive species interactions. Nature Communications 12: 6290.

Hiernaux P, Diarra L, Trichon V, Mougin E, Soumaguel N, Baup F. 2009. Woody plant population dynamics in response to climate changes from 1984 to 2006 in Sahel (Gourma, Mali). Journal of Hydrology 375: 103–113.

Jacobsen AL, Pratt RB. 2018. Extensive drought‐associated plant mortality as an agent of type‐conversion in chaparral shrublands. New Phytologist 219: 498–504.

Kane J, Meinhardt K, Chang T, Cardall B, Michalet R, Whitham T. 2011. Drought-induced mortality of a foundation species (*Juniperus monosperma*) promotes positive afterlife effects in understory vegetation. Plant Ecology 212: 733–741.

Keeley JE, Bond WJ, Bradstock RA, Pausas JG, Rundel PW. 2011. Fire in the Cape Region of South Africa. Pages 168–200 in. Fire in Mediterranean ecosystems: ecology, evolution and management. Cambridge University Press.

Le Maitre D, Midgley J. 1992. Plant reproductive ecology. Pages 135–174 in Cowling RM, ed. The ecology of fynbos: Nutrients, fire and diversity. Oxford University Press.

Le Maitre DC, Gaertner M, Marchante E, Ens E, Holmes PM, Pauchard A, O’Farrell PJ, Rogers AM, Blanchard R, Blignaut J. 2011. Impacts of invasive Australian acacias: implications for management and restoration. Diversity and Distributions 17: 1015–1029.

Lebel T, Ali A. 2009. Recent trends in the Central and Western Sahel rainfall regime (1990–2007). Journal of hydrology 375: 52–64.

Li H, Wang C, Yu Q, Smith E. 2022. Spatiotemporal assessment of potential drivers of salt marsh dieback in the North Inlet-Winyah Bay estuary, South Carolina (1990–2019). Journal of Environmental Management 313: 114907.

Li R, Zhu S, Chen HY, John R, Zhou G, Zhang D, Zhang Q, Ye Q. 2015. Are functional traits a good predictor of global change impacts on tree species abundance dynamics in a subtropical forest? Ecology Letters 18: 1181–1189.

Lloret F, Batllori E. 2021. Climate-induced global forest shifts due to heatwave-drought. Pages 155–186 in Canadell JG and Jackson RB, eds. Ecosystem Collapse and Climate Change. Springer.

Lucht W, Schaphoff S, Erbrecht T, Heyder U, Cramer W. 2006. Terrestrial vegetation redistribution and carbon balance under climate change. Carbon balance and management 1: 1–7.

Malhi Y, Aragão LE, Galbraith D, Huntingford C, Fisher R, Zelazowski P, Sitch S, McSweeney C, Meir P. 2009. Exploring the likelihood and mechanism of a climate-change-induced dieback of the Amazon rainforest. Proceedings of the National Academy of Sciences 106: 20610–20615.

Mann DH, Rupp TS, Olson MA, Duffy PA. 2012. Is Alaska’s boreal forest now crossing a major ecological threshold? Arctic, Antarctic, and Alpine Research 44: 319–331.

Marsh A, Blum LK, Christian RR, Ramsey E, Rangoonwala A. 2016. Response and resilience of *Spartina alterniflora* to sudden dieback. Journal of Coastal Conservation 20: 335–350.

Martínez-Vilalta J, Lloret F. 2016. Drought-induced vegetation shifts in terrestrial ecosystems: The key role of regeneration dynamics. Global and Planetary Change 144: 94–108.

McKee KL, Mendelssohn IA, D. Materne M. 2004. Acute salt marsh dieback in the Mississippi River deltaic plain: A drought‐induced phenomenon? Global Ecology and Biogeography 13: 65–73.

Mueller RC, Scudder CM, Porter ME, Talbot Trotter III R, Gehring CA, Whitham TG. 2005. Differential tree mortality in response to severe drought: evidence for long‐term vegetation shifts. Journal of Ecology 93: 1085–1093.

Mustart P, Rebelo A, Juritz J, Cowling R. 2012. Wide variation in post-emergence desiccation tolerance of seedlings of fynbos proteoid shrubs. South African Journal of Botany 80: 110–117.

Park IW, Hooper J, Flegal JM, Jenerette GD. 2018. Impacts of climate, disturbance and topography on distribution of herbaceous cover in Southern California chaparral: Insights from a remote‐sensing method. Diversity and Distributions 24: 497–508.

Park IW, Jenerette GD. 2019. Causes and feedbacks to widespread grass invasion into chaparral shrub dominated landscapes. Landscape Ecology 34: 459–471.

Phillips OL, Aragão LE, Lewis SL, Fisher JB, Lloyd J, López-González G, Malhi Y, Monteagudo A, Peacock J, Quesada CA. 2009. Drought sensitivity of the Amazon rainforest. Science 323: 1344–1347.

Pratt RB. 2022. Vegetation‐type conversion of evergreen chaparral shrublands to savannahs dominated by exotic annual herbs: causes and consequences for ecosystem function. American Journal of Botany 109: 9–28.

Pratt RB, Jacobsen AL, Ramirez AR, Helms AM, Traugh CA, Tobin MF, Heffner MS, Davis SD. 2014. Mortality of resprouting chaparral shrubs after a fire and during a record drought: physiological mechanisms and demographic consequences. Global Change Biology 20: 893–907.

Ratajczak Z, Ladwig LM. 2019. Will climate change push grasslands past critical thresholds? Pages 98–114 in Gibson DJ and Newman JA, eds. Grasslands and Climate Change. Cambridge University Press Cambridge, UK.

Redmond MD, Cobb NS, Clifford MJ, Barger NN. 2015. Woodland recovery following drought‐induced tree mortality across an environmental stress gradient. Global Change Biology 21: 3685–3695.

Redmond MD, Weisberg PJ, Cobb NS, Clifford MJ. 2018. Woodland resilience to regional drought: Dominant controls on tree regeneration following overstorey mortality. Journal of Ecology 106: 625–639.

Rigling A, Bigler C, Eilmann B, Feldmeyer‐Christe E, Gimmi U, Ginzler C, Graf U, Mayer P, Vacchiano G, Weber P. 2013. Driving factors of a vegetation shift from Scots pine to pubescent oak in dry Alpine forests. Global Change Biology 19: 229–240.

Rolando Jl, Hodges M, Garcia Kd, Krueger G, Williams N, Carr Jr J, Robinson J, George A, Morris J, Kostka Je. 2023. Restoration and resilience to sea level rise of a salt marsh affected by dieback events. Ecosphere 14: e4467.

Silliman BR, Van de Koppel J, Bertness MD, Stanton LE, Mendelssohn IA. 2005. Drought, snails, and large-scale die-off of southern US salt marshes. Science 310: 1803–1806.

Slingsby JA, Merow C, Aiello-Lammens M, Allsopp N, Hall S, Kilroy Mollmann H, Turner R, Wilson AM, Silander Jr JA. 2017. Intensifying postfire weather and biological invasion drive species loss in a Mediterranean-type biodiversity hotspot. Proceedings of the National Academy of Sciences 114: 4697–4702.

Stampfli A, Zeiter M. 2004. Plant regeneration directs changes in grassland composition after extreme drought: a 13-year study in southern Switzerland. Journal of Ecology 568–576.

Stampfli A, Zeiter M. 2008. Mechanisms of structural change derived from patterns of seedling emergence and mortality in a semi‐natural meadow. Journal of Vegetation Science 19: 563–574.

Suarez ML, Kitzberger T. 2008. Recruitment patterns following a severe drought: long-term compositional shifts in Patagonian forests. Canadian Journal of Forest Research 38: 3002–3010.

Suarez ML, Kitzberger T. 2010. Differential effects of climate variability on forest dynamics along a precipitation gradient in northern Patagonia. Journal of Ecology 98: 1023–1034.

Suarez ML, Sasal Y. 2012. Drought-induced mortality affects understory vegetation: release after death. Ecological Research 27: 715–724.

Toot R, Frelich LE, Butler EE, Reich PB. 2020. Climate-biome envelope shifts create enormous challenges and novel opportunities for conservation. Forests 11: 1015.

Trichon V, Hiernaux P, Walcker R, Mougin E. 2018. The persistent decline of patterned woody vegetation: The tiger bush in the context of the regional Sahel greening trend. Global Change Biology 24: 2633–2648.

Venturas MD, MacKinnon ED, Dario HL, Jacobsen AL, Pratt RB, Davis SD. 2016. Chaparral shrub hydraulic traits, size, and life history types relate to species mortality during California’s historic drought of 2014. PloS one 11: e0159145.

Vilà-Cabrera A, Martínez-Vilalta J, Galiano L, Retana J. 2013. Patterns of forest decline and regeneration across Scots pine populations. Ecosystems 16: 323–335.

Vilà-Cabrera A, Martínez-Vilalta J, Vayreda J, Retana J. 2011. Structural and climatic determinants of demographic rates of Scots pine forests across the Iberian Peninsula. Ecological Applications 21: 1162–1172.

Wendling V, Peugeot C, Mayor AG, Hiernaux P, Mougin E, Grippa M, Kergoat L, Walcker R, Galle S, Lebel T. 2019. Drought-induced regime shift and resilience of a Sahelian ecohydrosystem. Environmental Research Letters 14: 105005.

van Wilgen NJ, Goodall V, Holness S, Chown SL, McGeoch MA. 2016. Rising temperatures and changing rainfall patterns in South Africa’s national parks. International Journal of Climatology 36: 706–721.

Wunderling N, Staal A, Sakschewski B, Hirota M, Tuinenburg OA, Donges JF, Barbosa HM, Winkelmann R. 2022. Recurrent droughts increase risk of cascading tipping events by outpacing adaptive capacities in the Amazon rainforest. Proceedings of the National Academy of Sciences 119: e2120777119.

Zhou G, Houlton BZ, Wang W, Huang W, Xiao Y, Zhang Q, Liu S, Cao M, Wang X, Wang S. 2014. Substantial reorganization of China’s tropical and subtropical forests: based on the permanent plots. Global Change Biology 20: 240–250.

Zhou G, Peng C, Li Y, Liu S, Zhang Q, Tang X, Liu J, Yan J, Zhang D, Chu G. 2013. A climate change‐induced threat to the ecological resilience of a subtropical monsoon evergreen broad‐leaved forest in Southern China. Global Change Biology 19: 1197–1210.

Zhou G, Wei X, Wu Y, Liu S, Huang Y, Yan J, Zhang D, Zhang Q, Liu J, Meng Z. 2011. Quantifying the hydrological responses to climate change in an intact forested small watershed in Southern China. Global Change Biology 17: 3736–3746.
